# A Guide to Chatbots for COVID-19 Screening at Pediatric Health Care Facilities

**DOI:** 10.2196/18808

**Published:** 2020-04-30

**Authors:** Juan Espinoza, Kelly Crown, Omkar Kulkarni

**Affiliations:** 1 Department of Pediatrics Children's Hospital Los Angeles Los Angeles, CA United States; 2 Innovation Studio Children's Hospital Los Angeles Los Angeles, CA United States

**Keywords:** chatbots, COVID-19: pediatrics, digital health, screening

## Abstract

The coronavirus disease 2019 (COVID-19) outbreak has required institutions to rapidly adapt to changing public health circumstances. The Centers for Disease Control and Prevention has encouraged health care facilities to explore novel health care delivery modes. However, many institutions may not be prepared to begin offering digital health and telehealth services. Chatbots are one digital health tool that can help evolve triage and screening processes in a scalable manner. Here, we present a decision-making and implementation framework for deploying COVID-19 screening chatbots at pediatric health care facilities.

## Introduction

The International Health Regulations Emergency Committee of the World Health Organization (WHO) declared that the outbreak of novel coronavirus SARS‐CoV-2 (coronavirus disease 2019 or COVID-19) was a “public health emergency of international concern,” on January 30, 2020 [1]. Within 6 weeks, on March 11th, the WHO declared COVID-19 a global pandemic, defined as the worldwide spread of a new disease to which most people are susceptible [2,3]. In the United States, the number of cases has rapidly grown; there are more cases here than in any other country in the world [4,5].

The Centers for Disease Control and Prevention (CDC) has created interim guidance for health care facilities to address community transmission of COVID-19 [6]. The role of this guidance is to “reduce morbidity and mortality, minimize disease transmission, protect healthcare personnel, and preserve healthcare system functioning” [6]. These include recommendations around patient screening, working closely with public health agencies, creating contingency plans, monitoring health care workers, and managing ill patients at home when possible. Of note, the CDC recommends shifting health care delivery to remote options, such as phone management and telehealth. Given that approximately 80% of affected patients have mild symptoms, remote management can provide adequate care, though careful triage and frequent monitoring will be necessary [7]. Adoption of telehealth and digital health strategies has steadily progressed over the past 10 years, but is still not widespread, primarily due to provider/payer issues rather than technical ones [8,9]. Adopting the CDC recommendations may be difficult for institutions that do not have the innovation or information technology (IT) infrastructure necessary to either ramp up or deploy new services like telehealth. As such, it is important to triage patients appropriately so as not to overload newer programs.

One useful tool for patient triage are chatbots. Chatbots are applications that provide information or services through conversation-like interactions with users [10]. The underlying infrastructure can range from true artificial intelligence with natural language processing to simple conditional logic schemes with predetermined answers, similar to an online survey or quiz. Chatbots have become ubiquitous in retail and customer service but have only recently started expanding into health care [11]. Despite being relatively new, chatbots have already been adapted for a broad range of purposes in health care, including patient triage, clinical decision support for provider, directing patients and staff to appropriate resources, and even mental health applications, such as cognitive behavioral therapy and suicide interventions [12,13]. At least one review of the literature found that there is evidence that chatbots are both clinically effective and cost-effective [14].

Because chatbots can be deployed across email, web, social media, and text, they are an ideal tool to reach a large number of people in a short period of time. For COVID-19 and other infectious disease outbreaks, chatbots can screen patients, provide education, and triage patients to the right health care option (in-person appointment, telephone triage, telehealth appointment). One example of this is Providence Health, which deployed a COVID-19 chatbot built on a Microsoft platform to help triage patients to their telehealth services [15]. Here, we present a practical framework to help pediatric institutions think through the decision to leverage chatbot screening tools during the COVID-19 outbreak.

## Pediatric-Specific Considerations

Pediatric institutions face unique challenges during this pandemic. While children seem to be less likely to be infected and have milder symptoms, they are in most circumstances living with adults who may have very different exposures and medical risk factors than the child in question [7,16,17]. Moreover, when pediatric patients seek medical care, they are typically accompanied by an adult. As such, screening for signs, risk factors, and symptoms needs to address both the child and adults with whom the child is in close contact. It should be noted that the decreased severity of COVID-19 in pediatric patients is not uniform; very young infants and children with medical complexity are at increased risk, and as these groups are more likely to seek out care at pediatric institutions, appropriate precautions should still be taken [18,19].

Reducing crowd density and social distancing can be difficult at pediatric institutions. Children can be anywhere along the neurodevelopmental spectrum, age appropriate or delayed, and may struggle with key concepts like social distancing. Children are almost always accompanied by at least one adult, and it is not uncommon for one patient to be accompanied by two parents and siblings, and even grandparents. Chatbots can help address this by directing patients to the appropriate care modality early on and providing families with information relevant to their in-person appointments, such as visitor restrictions.

Finally, there is the issue of consent and access to medical care for children under the age of 18 years. In the United States, access to and consent for care without a parent or guardian varies from state to state. If the chatbot is widely accessible, institutions should develop protocols in collaboration with their legal and compliance teams to respond to chatbot users under the age of 18 years who either reach out for care or who screen as being high-risk.

## A Decision-Making Framework

Though it may seem daunting at first, deploying a health care screening chatbot can be a relatively fast process if key decisions can be clearly articulated along the way:

- Define the goal of your chatbot
- Identify the tools available for your chatbot to achieve its goal
- Choose a screening approach
- Access and distribution
- Buy versus build

### Define the Goal of Your Chatbot

For rapid deployment, such as during the current pandemic, chatbots work best when they have a simple and singular goal targeted for a specific user. This should be defined in one sentence (eg, “provide screening and education to the general public,” “educate and guide campus visitors on our current visitor restrictions,” or “triage existing patients to the right resources.”). All other decisions around technical infrastructure, workflows, and language choice will be guided from the driving purpose of the chatbot and the intended user.

### Identify the Tools Available for Your Chatbot to Achieve Its Goal

After screening, the chatbot will offer the user something based on their screening results (ie, information, a phone number to call, a call back, etc). These are the tools the chatbot uses to achieve its goal. Institutions should clearly define what the chatbot can and cannot offer. The possible options can be thought of in three categories:

1. The chatbot can provide information that the user can act on, either in the chat window or by directing the user to other resources (user-initiated). If the goal is to provide general information, the educational materials should be sourced from reputable sources like the CDC and other public health agencies. If the goal of the chatbot is to connect patients to specific resources, this can be done by providing the user a phone number, email, web address, etc, to reach out to request that service.
2. The chatbot can hand the user off to a human agent who can take a specific action (provider-initiated). This can be done in real time, by transitioning the conversation to a staff member, or by collecting contact information from the user, and then having staff members call the user back at a later time.
3. The chatbot can trigger an action in another software or system, such as appointment scheduling or medication refills (system-initiated). This can be the most technically challenging option and is likely not well suited for rapid deployment in a crisis situation.

User-initiated solutions are easier and faster to implement, can usually be incorporated into existing workflows, and have fewer data governance, legal, and compliance issues since there is no need to exchange Protected Health Information (PHI)/Personally Identifiable Information (PII). However, this type of solution may lead to situations in which a high-risk person still walks into a health care facility because they did not or could not follow through with the recommendations. Provider-initiated solutions allow providers to “close the loop” and can feel more personal. They ensure that facilities can be more proactive in properly addressing high-risk patients, but they require new workflows (and therefore additional staff, or additional work for existing staff), are typically more costly, and carry additional legal and compliance considerations. Finally, this decision can help inform whether you should deploy your chatbot inside or outside your institution's secure IT infrastructure.

### Choose a Screening Approach

The majority of chatbot interactions are a series of question-response dyads, with the user either free typing a response or choosing one from preselected options. Your chatbot should include a standard legal disclaimer and eligibility verification (“Are you a patient of…” or “What state/county/city do you live in?”), if relevant to the goal of the chatbot. In terms of screening for COVID-19, it is critical that institutions use the most up-to-date recommendations from trusted public agencies like the CDC. COVID-19 screening can be broken down into exposure risks, symptoms, comorbidities, and other risk factors (Table 1). Additional questions may be appropriate for your particular situation and what the follow-up to the chatbot interactions is meant to be. Institutions should be prepared to update all questions frequently as the situation develops and new criteria become relevant.

**Table 1:** Chatbot question structure.

Category	Sample question(s)	Notes
Disclaimer	“If you are experiencing an emergency…” Terms of use Privacy policy	Consult with your legal and compliance team on exact language/wording
Eligibility	Are you a patient of ____? Do you live in (city/county/state)? Do you have an upcoming appointment?	Eligibility criteria depend on institutional policy and preference
Exposures	Have you recently travelled to (list of countries)? Have you been in contact with someone who has travelled to these countries and is now sick? Have you been in contact with someone known to have coronavirus (COVID-19)? Have you been told by a public health official that you may have been exposed to coronavirus (COVID-19)?	Follow recommendations of the CDC^a^ and public health agencies, and update frequently Include language to address children and their adult close contacts
Symptoms	Are you experiencing any of the following symptoms?	Follow recommendations of the CDC and public health agencies, and update frequently
Risk factors	Do you have any of the following health conditions?	Follow recommendations of the CDC and public health agencies, and update frequently
Follow-up	Do you have access to…? Are you interested in having a telehealth visit? Please provide a call back number/email.	These questions are dependent on your institution's follow-up plan for chatbot interactions

^a^CDC: Centers for Disease Control and Prevention.

### Access and Distribution

Chatbots are capable of interacting with users in a variety of ways. They can be deployed natively in social media platforms, via text messages, email, and embedded in a web page. Native deployment creates a smoother user experience but requires more work and can be more costly. Deploying the chatbot on the web and redirecting users via links distributed through multiple channels (social media, email, text messages) can be more cost-effective and simpler to manage. You should make this decision based on how you will reach as many of your intended users as possible. You can consider the current utilization rates of various communication services you already offer patients and families to help make this decision. For example, if you have very few patients enrolled in your portal, then that is unlikely to be a good deployment strategy. However, if you use text reminders for appointments and families frequently reply back to confirm, then text message may be a better choice.

### Buy Versus Build

For institutions with the right technical expertise, there are several frameworks that can be used to build your own chatbot, at little or no cost. For others, several companies provide low-cost chatbot services that include design, implementation, maintenance, and data reporting. Very importantly, note that if your chatbot collects PHI/PII or is integrated into a hospital information system, it will typically require legal and compliance review, along with information security assessment and (likely) a business associate’s agreement (BAA). A BAA is a contract between a covered entity (a health care provider) and a third party that accesses PHI in order to perform a service for the covered entity and is required to be in compliance with the Health Insurance Portability and Accountability Act (HIPAA) [20]. If your institution has an existing BAA with an information services company like Microsoft, Amazon, or Google, then leveraging their chatbot services may be the most expedient way to deploy a solution.

## Implementation

After completing the decision-making exercise described in the previous section, the next phase of chatbot deployment is implementation. We recommended using a human-centered design approach and starting with your user experience and journey [21,22]. Technology experiences designed with the user in mind are more likely to be engaging and effective.

### Map the User Experience and Workflows

**Figure 1:**
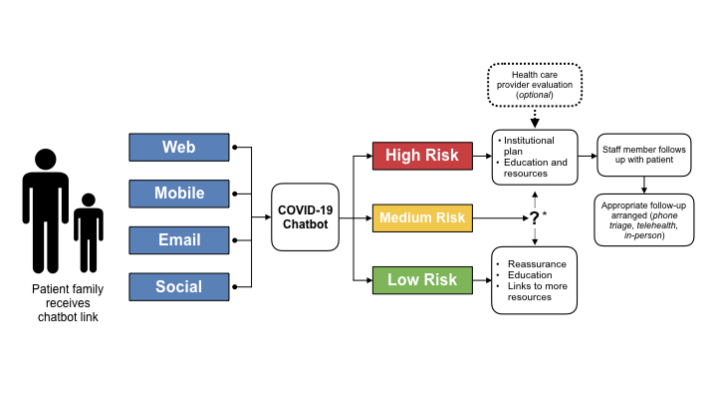
Sample chatbot process map. *: Institutional discretion, follow public health agency guidelines.

Implementation is the make-or-break of any digital health project, and chatbots are no different. A valuable exercise is to make process maps and storyboard the experience for both the users and the providers that will interact with the chatbot. This is equally effective in low fidelity (eg, post-it notes, whiteboards) and high fidelity (eg, slide decks, animations) formats. A sample process map can be seen in Figure 1. In this example, the family might receive a link to the chatbot through the web, text, email, or social media, and then engage with the chatbot. The chatbot would administer the screening questions and then triage the patient into a risk category, each with a specific set of actions.

### Chatbot Architecture

**Figure 2:**
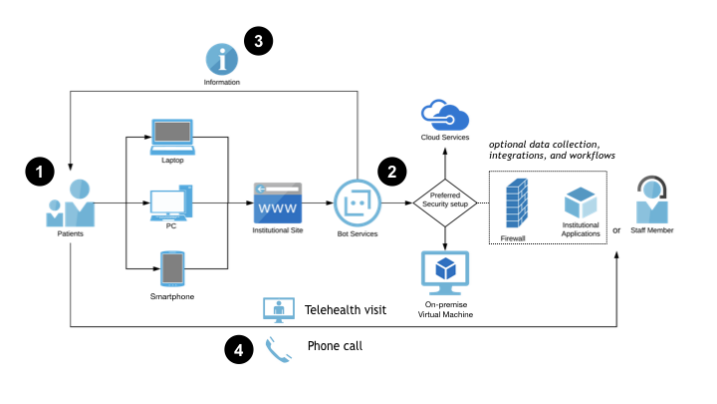
A chatbot network diagram: patient-initiated approach. This diagram illustrates a generic architecture and workflow for a simple chatbot that is hosted outside the institution’s secure computing environment. 1) Patient uses a personal device to access the institution’s website from a link they receive in a text, email, or secure message. 2) The chatbot interacts with the patient and stratifies them according to the screening algorithm. 3) The chatbot provides the patient with education, resources, tips, and specific instructions on next steps. 4) The patient reaches out to the hospital via phone or telehealth as instructed by the chatbot. Integration with hospital systems is possible for data collection and workflow management, but not required.

**Figure 3:**
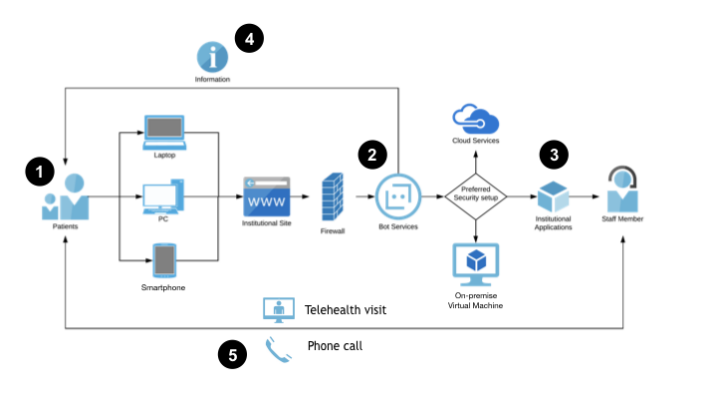
A chatbot network diagram: provider-initiated approach. This diagram illustrates a generic architecture and workflow for a chatbot that is hosted within the institution’s secure computing environment. 1) The patient uses a personal device to access the institution’s website from a link they receive in a text, email, or secure message. 2) The chatbot interacts with the patient and stratifies them according to the screening algorithm. 3) The chatbot collects data and integrates with institutional workflows, including additional clinical screening and scheduling. 4) The chatbot provides the patient with education, resources, tips, and specific instructions on next steps. 5) Staff members reach out to patients to schedule appropriate follow-up; patients are also able to reach out with further questions or requests.

Once you have defined your ideal user experience, you can begin to explore how that chatbot integrates into your IT ecosystem. The exact specifications will be highly dependent on the decisions you made regarding goals, users, actions, and workflows. For example, a chatbot that provides users with informational resources based on their risk stratification, but does not collect any PHI/PII, may not require any data storage or tracking and could be run completely from a cloud-based web service. On the other hand, if you will be collecting PHI/PII, or connecting users to other secure hospital information systems like a patient portal, you will require a secure, HIPAA-compliant environment. Figures 2 and 3 provide two network diagram examples of how this might be set up in your local environment. In Figure 2, we see a straightforward, patient-initiated approach, where the patient interacts with a web-hosted bot that provides the patient with information that they can act on, such as calling a hotline for an appointment or logging into a telehealth portal. By contrast, the chatbot in Figure 3 is located in a secure environment (in this case, behind an institutional firewall), enabling it to collect PHI/PII and integrate into other institutional applications. Staff members can act on the collected data, while the patient is provided with appropriate education and resources.

### Chatbot Data

Chatbots generate a significant amount of data and metadata, including user volumes, timestamps, length of conversations, user responses, and link tracking. Your institution will need to decide which data, if any, you are interested in tracking and analyzing. It may be worthwhile to collect chatbot data even if no PII/PHI is being tracked; modern informatics techniques have been used to track the spread of infectious diseases by analyzing social media posts as well as search engine queries; chatbot interactions might also be a useful source of public health data [23,24].

### Testing Your Chatbot

User testing is critical prior to launch. Identify a small group of individuals who will spend time trying to “break” the tool. The goal of user testing is to discover everything that is wrong with the product you built. Your users should be looking for problems in the text, nonsequiturs in the workflow, broken links, bad phone numbers, typos, etc. This is also an opportunity to verify that your database is collecting information in the way it was designed. Once you have completed several iterative rounds of testing, you are ready for deployment to patients.

## Conclusions

Chatbots are a low-cost tool that can be deployed rapidly to screen large numbers of patients before they go to a health care facility. However, there are several variables that require careful consideration. With the right design, they can provide users with appropriate education and information, and triage patients to alternative health care delivery models like telephone triage and telehealth appointments. As institutions become more familiar with these tools, they can be repurposed in the future for other public health emergencies, as well as for more standard care uses. We hope that this framework for decision making and implementation is helpful to others; our Innovation Studio team is available to support any institution considering deploying similar tools.

## Abbreviations

BAA: business associate’s agreement
CDC: Centers for Disease Control and Prevention
COVID-19: coronavirus disease 2019
HIPAA: Health Insurance Portability and Accountability Act
IT: information technology
PHI: Protected Health Information
PII: Personally Identifiable Information
WHO: World Health Organization

## References

[1] (2020). World Health Organization.

[2] (2020). World Health Organization.

[3] (2010). World Health Organization.

[4] (2020). Centers for Disease Control and Prevention.

[5] (2020). Johns Hopkins Coronavirus Resource Center.

[6] (2020). Centers for Disease Control and Prevention.

[7] Wu Z, McGoogan JM (2020). Characteristics of and Important Lessons From the Coronavirus Disease 2019 (COVID-19) Outbreak in China: Summary of a Report of 72 314 Cases From the Chinese Center for Disease Control and Prevention. JAMA.

[8] Olson CA, McSwain SD, Curfman AL, Chuo J (2018). The Current Pediatric Telehealth Landscape. Pediatrics.

[9] Barnett ML, Ray KN, Souza J, Mehrotra A (2018). Trends in Telemedicine Use in a Large Commercially Insured Population, 2005-2017. JAMA.

[10] Roca S, Sancho J, García J, Alesanco A (2020). Microservice chatbot architecture for chronic patient support. J Biomed Inform.

[11] Palanica A, Flaschner P, Thommandram A, Li M, Fossat Y (2019). Physicians' Perceptions of Chatbots in Health Care: Cross-Sectional Web-Based Survey. J Med Internet Res.

[12] Montenegro JLZ, da Costa CA, da Rosa Righi R (2019). Survey of conversational agents in health. Expert Systems with Applications.

[13] Laranjo L, Dunn AG, Tong HL, Kocaballi AB, Chen J, Bashir R, Surian D, Gallego B, Magrabi F, Lau AYS, Coiera E (2018). Conversational agents in healthcare: a systematic review. J Am Med Inform Assoc.

[14] Andersson G (2016). Internet-Delivered Psychological Treatments. Annu Rev Clin Psychol.

[15] (2020). Providence Health & Services.

[16] Velavan TP, Meyer CG (2020). The COVID-19 epidemic. Trop Med Int Health.

[17] Li Q, Guan X, Wu P, Wang X, Zhou L, Tong Y, Ren R, Leung KSM, Lau EHY, Wong JY, Xing X, Xiang N, Wu Y, Li C, Chen Q, Li D, Liu T, Zhao J, Liu M, Tu W, Chen C, Jin L, Yang R, Wang Q, Zhou S, Wang R, Liu H, Luo Y, Liu Y, Shao G, Li H, Tao Z, Yang Y, Deng Z, Liu B, Ma Z, Zhang Y, Shi G, Lam TTY, Wu JT, Gao GF, Cowling BJ, Yang B, Leung GM, Feng Z (2020). Early Transmission Dynamics in Wuhan, China, of Novel Coronavirus-Infected Pneumonia. N Engl J Med.

[18] Dong Y, Mo X, Hu Y, Qi X, Jiang F, Jiang Z, Tong S (2020). Epidemiology of COVID-19 Among Children in China. Pediatrics.

[19] Coleman C, Kuo, Dennis (2020). Healthychildren.org.

[20] (2013). Department of Health and Human Services.

[21] Altman M, Huang TTK, Breland JY (2018). Design Thinking in Health Care. Prev Chronic Dis.

[22] Roberts JP, Fisher TR, Trowbridge MJ, Bent C (2016). A design thinking framework for healthcare management and innovation. Healthc (Amst).

[23] Jahanbin K, Rahmanian F, Rahmanian V, Jahromi AS (2019). Application of Twitter and web news mining in infectious disease surveillance systems and prospects for public health. GMS Hyg Infect Control.

[24] Verma M, Kishore K, Kumar M, Sondh AR, Aggarwal G, Kathirvel S (2018). Google Search Trends Predicting Disease Outbreaks: An Analysis from India. Healthc Inform Res.

